# The American Medical Association

**Published:** 1855-04

**Authors:** 


### EDITORIAL.

*The American Medical Association.*

The Eighth Annual Meeting of the American Medical Association will be held in the city of Philadelphia, on Tuesday, May 1, 1855.

The Secretaries of all the Societies and other bodies entitled to representation in the Association, are requested to forward to the undersigned correct lists of their respective delegations *as soon as they may be appointed;* and it is *earnestly* desired by the Committee of Arrangements that the appointments be made at as early a period as possible.

The following are extracts from Article 2d of the Constitution:

"Each local Society shall have the privilege of sending one delegate for every ten of its regular resident members, and one for every additional fraction of more than half of this number. The faculty of every regularly constituted medical college or chartered school of medicine, shall have the privilege of sending two delegates. The professional staff of every chartered or municipal hospital containing a hundred inmates or more, shall have the privilege of sending two delegates, and every other permanently organized medical institution of good standing shall have the privilege of sending one delegate.

"Delegates representing the medical staff of the United States army and navy shall be appointed by the Chiefs of Army and Navy Medical Bureau.

"The number of delegates so appointed shall be four from the army medical officers, and an equal number from the navy medical officers."

The latter clause, in relation to delegates from the army and navy, was adopted as an amendment to the constitution at the meeting of the Association, held in New York, in May, 1853.

FRANCIS WEST, M.D.,
One of the Secretaries, 352 Chestnut St., Philadelphia.

By the above notice of the Secretary, it will be seen that another Annual Meeting of the Association is near at hand.

Have all the Medical Societies and Institutions in the North-West appointed their delegates? If not, we hope they will do so without delay. The coming meeting will, doubtless, be one of more interest, in a strictly scientific and professional aspect, than any which have preceded. It is easy to perceive that the current of opinion in the profession is running strongly in favor of devoting more time and care to the reading and consideration of such scientific papers and reports as may be presented, and less to mere miscellaneous matters. Heretofore much time has been consumed on random resolutions and vain attempts to amend the constitution.

Let each delegate look over the list of subjects assigned to special committees, and study those subjects in such a manner as to be prepared to consider and act upon any reports that may be presented. We say this much without wishing to convey the impression that no time ought to be devoted to the important subject of *medical education*.

We do think, however, that fewer resolutions and more *practical action* on this subject would conduce to the interests of the profession. An effort will probably be made at the coming meeting to so far change the constitution as to permit the Annual Meetings to be held permanently in Washington city. We are very sure, however, that such a step would greatly lessen the influence and importance of the Association. If there is one single constitutional provision which has contributed largely to the influence of the national organization over the whole profession, it is that which prohibited it from meeting twice in succession in the same place. On this subject we hope the North-West will not only be well represented, but also well united in opposition to any change which shall aid to localize the Annual Meetings of the Association in one section of the Union.

